# Children’s fear and sleep: what is the relationship

**DOI:** 10.1192/j.eurpsy.2023.1504

**Published:** 2023-07-19

**Authors:** C. Pires-Lima, M. H. Figueiredo, V. Guedes

**Affiliations:** CINTESIS - Center for Health Technology and Services Research, Porto, Portugal

## Abstract

**Introduction:**

Fear was the primary construct for this study, however, knowing that high levels of anxiety can cause changes in sleep quality, it was considered opportune to study the relationship between these two concepts.

According to Marks (1969), “Fear is a normal response to active or imagined threat in higher animals, and comprises an external behavioral expression, an inner feeling and associated physiological changes”, almost all children experience some degree of fear during its development. Additionally, while these fears vary in frequency, intensity, and duration, they tend to be mild, age-specific, and transient.

According to a simple definition, sleep is a reversible behavioral state of perceptive disconnection and indifference to the environment (Carskadon & Dement, 1989).

**Objectives:**

Sleep quality perceived by children is inversely correlated with self-perception of fears

**Methods:**

Participants

The study sample consists of 121 students from the 1st cycle of basic education, 65 (53.7%) attending the 3rd year of schooling and 56 (46.3%) attending the 4th year of schooling, 66 ( 54.5%) were female and 55 (45.5%) were male, aged between 7 and 10 years old (M=8.5; SD=0.61).

**Method:**

Sleep Self Report-PT (SSR-PT): The SSR-PT is a questionnaire designed to assess children from 7 to 12 years old regarding their self-perception of the quality of their sleep. Fear Survey Schedule for Children-Revised (FSSC-R): T. Ollendick (1978); Translation and adaptation: Pedro Dias & Miguel Gonçalves

**Results:**

The lower the sleep quality perceived by the children, the greater the self-perception of fears of the two factors with very strong significance .000 in both.

**Conclusions:**

The lower the sleep quality perceived by the children, the greater the self-perception of fears of the two factors with very strong significance .000 in both.

**Disclosure of Interest:**

None Declared

